# Mirror Self-Recognition in Pigeons: Beyond the Pass-or-Fail Criterion

**DOI:** 10.3389/fpsyg.2021.669039

**Published:** 2021-05-17

**Authors:** Neslihan Wittek, Hiroshi Matsui, Nicole Kessel, Fatma Oeksuez, Onur Güntürkün, Patrick Anselme

**Affiliations:** ^1^Faculty of Psychology, Department of Biopsychology, Ruhr University Bochum, Bochum, Germany; ^2^Faculty of Psychology, University of Hagen, Hagen, Germany

**Keywords:** self-recognition, pigeons, movement synchronicity, DeepLabCut, behavior, foraging

## Abstract

Spontaneous mirror self-recognition is achieved by only a limited number of species, suggesting a sharp “cognitive Rubicon” that only few can pass. But is the demarcation line that sharp? In studies on monkeys, who do not recognize themselves in a mirror, animals can make a difference between their mirror image and an unknown conspecific. This evidence speaks for a gradualist view of mirror self-recognition. We hypothesize that such a gradual process possibly consists of at least two independent aptitudes, the ability to detect synchronicity between self- and foreign movement and the cognitive understanding that the mirror reflection is oneself. Pigeons are known to achieve the first but fail at the second aptitude. We therefore expected them to treat their mirror image differently from an unknown pigeon, without being able to understand that the mirror reflects their own image. We tested pigeons in a task where they either approached a mirror or a Plexiglas barrier to feed. Behind the Plexiglas an unknown pigeon walked at the same time toward the food bowl. Thus, we pitched a condition with a mirror-self and a foreign bird against each other, with both of them walking close toward the food bowl. By a detailed analysis of a whole suit of behavioral details, our results make it likely that the foreign pigeon was treated as a competitor while the mirror image caused hesitation as if being an uncanny conspecific. Our results are akin to those with monkeys and show that pigeons do not equal their mirror reflection with a conspecific, although being unable to recognize themselves in the mirror.

## Introduction

Spontaneous mirror self-recognition is limited to humans (starting with 15–24 month-old children) and a few other species, including chimpanzees, orangutans, elephants, dolphins, Indian house crows, and magpies (Gallup, 1970; Amsterdam, 1972; Suarez and Gallup, 1981; Anderson, 1984; Reiss and Marino, 2001; Rochat, 2003; Plotnik et al., 2006; Prior et al., 2008; Buniyaadi et al., 2019). According to a recent striking study, it even occurs in the cleaner wrasse fish (*Labroides dimidiatus*), which is capable of detecting a colored mark on its throat with the help of a mirror and subsequently displays throat-scraping behavior to remove the mark (Kohda et al., 2019). As in the fish example, the traditional mirror self-recognition (MSR) task uses a painted, body-attached or injected mark that cannot be seen without the help of a mirror. MSR is assumed to occur if an animal spontaneous attempts to inspect or remove the mark in front of a mirror (Gallup, 1970).

However, the mark test only produces binary pass-or-fail results, suggesting the existence of a sharp evolutionary ‘cognitive Rubicon’ that only a few species can pass (de Waal, 2019). But even within these species, many individuals fail to succeed (Povinelli et al., 1993; Prior et al., 2008; Buniyaadi et al., 2019). In addition, several experimental findings seem not to be consistent with an all-or-nothing interpretation of MSR. For instance, rhesus monkeys (*Macaca mulatta*) fail spontaneous MSR tasks, but start using mirrors to scrutinize their body after being trained on a refined version of the mark test (Chang et al., 2015, 2017; Huttunen et al., 2017). Moreover, although monkeys do not recognize their mirror image at a first glance, they are capable of using mirrors to locate hidden objects or unseen conspecifics (Eglash and Snowdon, 1983; Bayart and Anderson, 1985; Macellini et al., 2010). Capuchin monkeys (*Cebus apella*) also fail MSR but react to their mirror image as if it was an unreal, uncanny individual rather than a stranger or themselves (de Waal et al., 2005). These results provide evidence for an intermediate psychological state (or capability) between full MSR and its total absence (de Waal, 2019). Therefore, we hypothesize that the ability to detect the correlation between self and mirror movements is a necessary but insufficient component of self-recognition. The minimal further component is possibly the cognitive ability to conclude that movement synchronicity results from the fact that the reflected animal in the mirror is oneself. Species that detect the correlation but fall short of understanding the implication could constitute an intermediate group between pass and fail. They do not pass MSR but recognize that the animal in the mirror is somehow different from a conspecific. Thus, they should treat their reflection not like they would do when being confronted with an unknown conspecific. We set out to test this prediction using pigeons.

Pigeons do not show spontaneous MSR but can be conditioned to exhibit self-recognition-like behavior (Epstein et al., 1981; Uchino and Watanabe, 2014). Additionally, pigeons are able to detect synchronicity between their own and video-based movements (Toda and Watanabe, 2008). Thus, pigeons are ideal candidates to test our assumption that such animals should treat their mirror image different from an unknown pigeon, although not passing MSR. Our task differed from traditional MSR experiments in that we aimed to determine how exposure to a mirror image or an unknown conspecific behind a Plexiglas panel altered the approach and consumption of food as well as individual behavior. The assessment of MSR was therefore indirect and carried out in a context in which the animal is producing a natural and expected behavior (approaching food, followed by feeding). In addition to telling us how sensitive pigeons are to the presence of other pigeons competing for food, differences in foraging activity and other behavioral alterations between the two conditions (mirror vs. stranger) would indirectly suggest that they treat their reflection differently from another pigeon. By combining traditional measurements and the dynamical analysis of behavioral patterns, we showed that, like monkeys, pigeons do not perceive their mirror image as a stranger.

## Materials and Methods

### Animals and Housing Conditions

Sixteen naïve adult homing pigeons (*Columba livia*, 14 males and 2 females) obtained from local breeders were maintained at 85–90% of their free-feeding body weight throughout the experiment, while water was accessible *ad libitum*. Eight pigeons used as experimental pigeons were housed under visual isolation in individual cages. The other eight pigeons were unknown pigeons (strangers) housed inside an aviary under a 12 h light/dark cycle (lights on at 7:30 am). Thus, no social interaction occurred between the experimental and the unknown pigeons while housed. All procedures followed the German guidelines for the care and use of animals in science, and were in accordance with the European Communities Council Directive 86/609/EEC concerning the care and use of animals for experimentation.

### Apparatus

The experiment was conducted in a wooden box with two identical compartments (50 cm long × 60 cm width × 30 cm height). Depending on the experimental phase, either a gray opaque plastic wall, a transparent Plexiglas panel or a mirror of identical size (50 cm long × 30 cm height) was used to separate the compartments. A bowl (10.8 cm long × 5.3 cm width × 4.3 cm height) with 10 g of a grain mixture was located in front of the separation panel, in the corner opposite to the entrance and adjacent to the separation. Each session was recorded with an external camera (Sony Hybrid HDD). For *a posteriori* deep learning analysis, the training of the experimental pigeons was recorded by a GoPro (Hero4 Session) placed on top of the compartment.

### Conditions

We tested our birds under three conditions:

#### Approaching a Wall (Wall)

Pigeons approached a feeder placed at the front corner of a gray opaque plastic wall.

#### Approaching a Mirror (Mirror)

Pigeons approached a feeder placed at the front corner of a mirror.

#### Approaching an Unknown Pigeon (Stranger)

Pigeons approached a feeder placed at the front corner of a Plexiglas panel; behind this panel, an unknown pigeon was approaching its own feeder at the same time.

**Figure 1:**
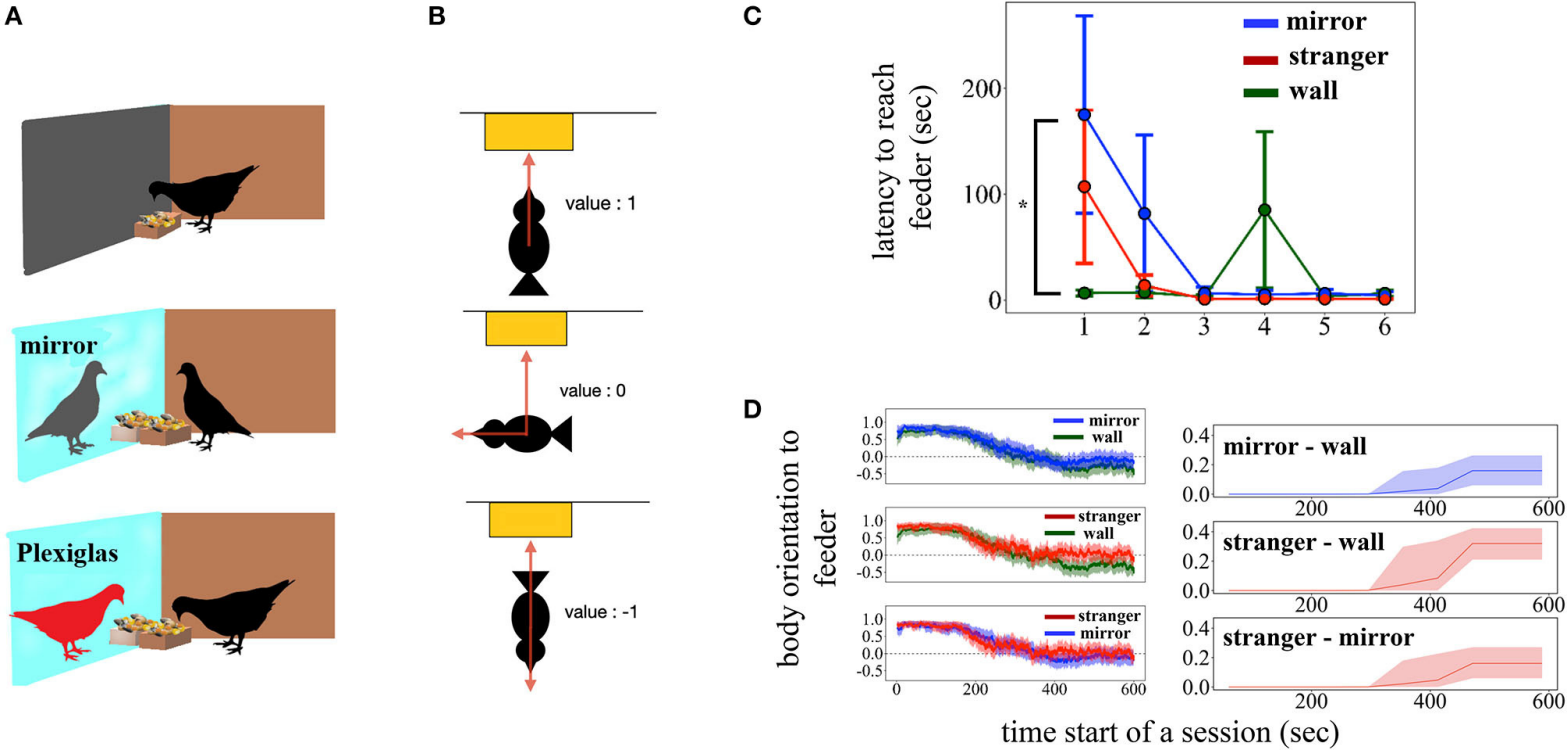
**(A)** Schematic illustration of the experimental setup in the Wall, Mirror and Stranger conditions, top to bottom, respectively. **(B)** Schematic illustration of the body orientation indexes. The value of 1 indicates a body orientation toward feeder/mirror, −1 is an orientation opposite to feeder/mirror, and 0 describes an orientation of 90° relative to feeder/mirror. **(C)** Latency to approach the feeder. **(D)** *Left*, mean body orientation indexes across time. Filled regions represent SEM. *Right*-time-series modeling revealed the differences between two conditions. Lines represent posterior mean and shaded areas 95% credible intervals. *p < 0.05.

The experimental pigeons were used in balanced order throughout the three conditions (Figure 1A). The stranger pigeons were only used as companions behind the Plexiglas panel in the third condition.

### Procedure

*Wall* and *Mirror* sessions started with the placement of a pigeon into a compartment through the entrance door from where it walked to the feeder. In the *Stranger* condition, one unknown pigeon and one experimental pigeon were placed in adjacent compartments through their separate entrance doors. Both pigeons walked toward their feeder, thereby inevitably approaching the Plexiglas from both sides. Thus, the *Mirror* and *Stranger* conditions only differed with the respect to the individual pigeon seen by the experimental bird. In the *Mirror* condition, it was itself, and in the *Stranger* condition an unknown conspecific. Half of the pigeons were tested in the left compartment and the other half in the right compartment to eliminate any side effects. Sessions involving a mirror or a Plexiglas panel were counterbalanced across the experimental pigeons, each of them being always tested with the same member (of the same sex) of the stranger pigeons. Pairs of pigeons were stable over time. This was justified by the fact that the experimental birds also got used to their mirror reflection over time. Depending on the session, the experimental birds could therefore only see the wall, their reflection (mirror) or the same stranger pigeon (Plexiglas) in the neighboring compartment.

To begin with, the experimental pigeons were habituated to the mirror in their home cage for five consecutive days with 1 h each day. This step was necessary to avoid initial arousal when the pigeons were exposed to a mirror for the first time (Gallup, 1970; Suarez and Gallup, 1981; Plotnik et al., 2006). Prior to this experiment, they had also been exposed to real individuals on many occasions since they had been raised in flocks. Then, all pigeons were habituated to the experimental box for two consecutive days with 10 min per day. After this habituation phase, the experimental pigeons were exposed to each condition (*Wall, Mirror*, and *Stranger*) for a total of 6 days or sessions per condition. Their presentation was counterbalanced across the experiment, which started and ended with an exposure to the *Wall* condition. Each session lasted 10 min and was video recorded (see Supplementary Material). Note that there was perfect synchronicity during approach and feeding between the pigeon and its reflection in the *Mirror* condition, while synchronicity was obviously lower in the *Stranger* condition.

### Behaviors Analyzed per Session

#### Time Latency to Reach the Feeder

Time elapsed between the placement of the pigeon in the arena and its first peck in the feeder.

#### Total Number of Pecks

Number of times the pigeon had a vertical head movement directed to the feeder. It was impossible to see if thereby a grain was ingested.

#### Body Orientation

Because of the pigeons' body posture during feeding, it was technically not possible to track their beak—which would have given direct information of their head orientation. So, body orientation was used as the closest indicator of their activity. It is the cosine of the body-to-head vector relative to the center of the feeder in front of either the wall, the Plexiglas partition, or the mirror. A value of 1 means that the pigeon is oriented straight to the feeder, 0 that the pigeon stands 90 degrees to it, and −1 that the pigeon turns its back to the feeder (Figure 1B). Note that these different orientations relative to the feeder also work with respect to the mirror self-image or the unknown pigeons behind the Plexiglas. In addition, due to the anatomy of the pigeon's skeleton, body orientation during feeding can be assumed to be similar to the head orientation.

#### Activity Rate

Average speed of body movement while not feeding. Feeding episodes were defined by the proximity of the head position of pigeons to the feeder using tracking protocol as described below. Speed was measured by position changes between subsequent frames and was then log-transformed for statistical analysis for normality.

#### Other Behaviors

Because our experiment reflected various aspects of social foraging, the videos were in addition manually checked in detail to detect any occurrence of individual behaviors as described below and shown in the Supplementary Videos:

##### Shaking

Moving of body parts like head, tail, or wing along a curve in fluent and repeated motion.

##### Preening

Maintenance behavior which involves the use of the beak to reposition feathers on different parts of the body. A preening event was scored each time the beak touched the body and was scored until its end.

##### Head Scratching

Left or right foot scratches the head.

##### Wing Flapping/Opening

Partial or total extension of one or both wings with occasional flapping.

##### Pecking Plexiglas/Mirror/Wall

Pecks at the barrier that separates the compartments.

##### Attack

The barrier separating the compartments is hit with part of the body without using the beak.

### Video Tracking and Statistical Analyses

Video-tracking was performed by using the machine-learning-based tracking software DeepLabCut (Nath et al., 2019), excluding the first 2 s of the videos from the analyses. This exclusion was required to normalize the data between conditions since the stranger pigeon was put into the box 1–2 s before the experimental pigeon. The tracked trajectories were smoothed using LOESS (locally estimated scatterplot smoothing) to remove noise. These tracked trajectories were used as input for body orientation and activity rate analysis. Body orientation over time was compared between conditions using a time series modeling technique. Briefly, the model incorporates dynamic fluctuations of body orientation, the difference between conditions, and the periods during which the difference occurred. These variables allowed us to examine the effects of the mirror and of the unknown pigeon, and the time periods during which those effects occurred. The difference was evaluated by checking whether Bayesian credible intervals overlapped with 0. Time latency to reach the feeder, total numbers of pecks, and the session effect within each condition to examine the familiarization of pigeons were compared by means of a linear mixed model. Individual differences were accounted for as random effects. The significance was tested with likelihood ratio tests, and the Tukey's test was used as a *post-hoc* multiple comparison method. Partial η^2^ was reported as an effect size.

Individual behavior analysis and classification were carried out by the experimenter and a blind observer who was naïve relative to the experimental questions. The experimenter trained the blind observer to identify the different behaviors of interest in this study. To determine inter-rater reliability, 44 of the 144 videos were scored by the experimenter and the 144 videos were scored by the blind observer. An interrater reliability analysis using the Kappa statistic confirmed the consistency among the experimenter and the blind observer (Kappa = 0.85, *p* < 0.001). A two-way repeated measures ANOVA was conducted to compare the effects of condition and day on the number of observed behaviors. For the comparison between conditions, a Tukey's test was used as a *post-hoc* test. Additionally, a repeated measures ANOVA was applied to examine the session effects within each condition. Statistical analysis was performed using R 3.5.3 (R Core Team, 2020) and multiple Python packages (McKinney, 2010; Harris et al., 2020; The Pandas Development Team, 2020).

## Results

The latency to reach the feeder decreased over sessions (Figure 1C; χ^2^(5) = 53.52, *p* < 0.0001, η_*p*_^2^ = 0.31), the main effect of each condition on latency fell just short of significance [χ^2^(2) = 5.98, *p* = 0.0503, η_*p*_^2^ = 0.05], and a significant Condition × Session interaction was found [χ^2^(10) = 26.56, *p* = 0.003, η_*p*_^2^ = 0.18]. A *post-hoc* comparison showed that the latency to reach the feeder was overall higher in the *Mirror* condition and the difference was mostly observable when compared to the *Stranger* condition (*Mirror* vs. *Stranger*: *z* = 2.28, pcorr = 0.058; *Mirror* vs. *Wall*: *z* = 1.41, pcorr = 0.33, *Stranger* vs. *Wall*: *z* = 0.87, pcorr = 0.66). The latency in the *Mirror* condition was the highest and differed significantly from the *Wall* condition in the first session (*z* = 3.22, pcorr = 0.004). The overall comparison of the sessions within the conditions revealed that the session effect was significant in the *Stranger* and the *Mirror* conditions, while this effect was not significant in the *Wall* condition [*Stranger*: χ^2^(5) = 38.12, *p* < 0.0001, *Mirror*: χ^2^(5) = 38.80, *p* < 0.0001, *Wall*: χ^2^(5) = 4.21, *p* = 0.520]. Further *post-hoc* tests showed that only the 1st session was significantly different from the other sessions in the *Mirror* and the *Stranger* conditions, while performance remained similar in the rest of the sessions (session 1 vs. other sessions; *Stranger*: pcorr ≤ 0.05, *Mirror*: pcorr ≤ 0.02). The total number of pecks were not significantly different across conditions [χ^2^(2) = 5.87, *p* = 0.053, η_*p*_^2^ = 0.05] and sessions [χ^2^(5) = 5.67, *p* = 0.34, η_*p*_^2^ = 0.05].

The time series model revealed that both the *Mirror* and the *Stranger* conditions evoked behavior that was more oriented toward the feeder and hence toward the other bird (Figure 1D), upper and middle panels; posterior mean 0.16 (9.17 degrees) with a 95% CI (0.06 0.26) between *Wall* and *Mirror*; 0.32 (18.66 degrees) with (0.21 0.42) between *Wall* and *Stranger*. The amount of feeder/bird-directed behavior was higher in the *Stranger* than in the *Mirror* condition according to a 95% CI (Figure 1D), bottom panel; −0.16 (−9.24 degrees) with [−0.27 −0.06], indicating that the real conspecific individual grabbed more attention.

**Figure 2:**
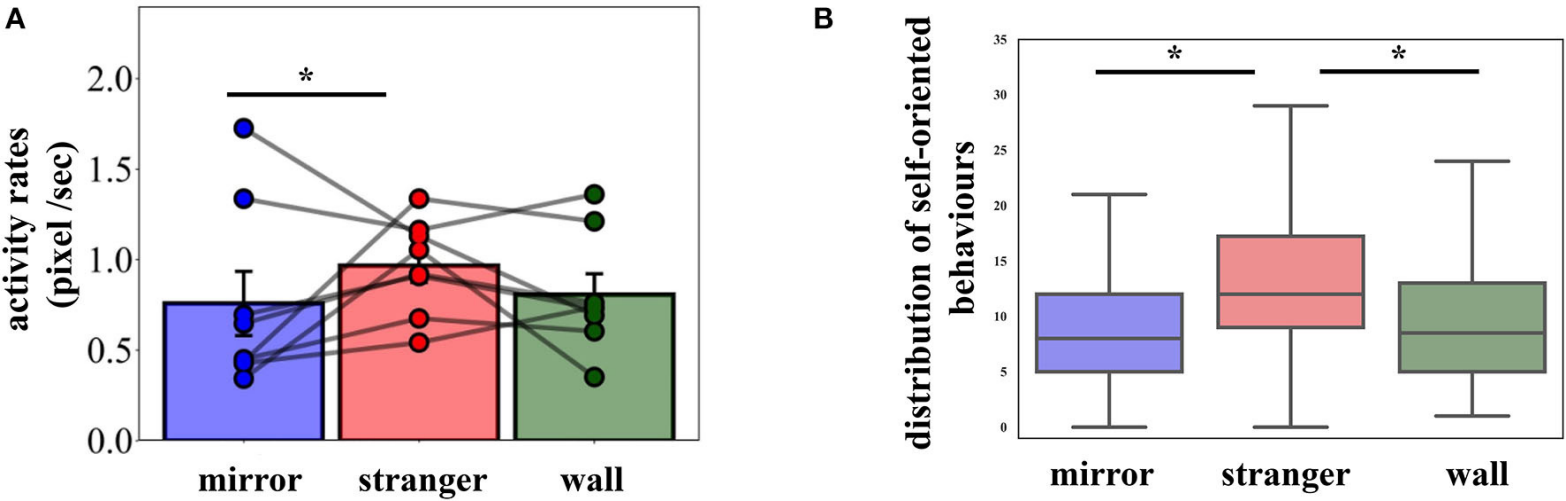
**(A)** Mean activity rates with the individual data points. Error bars represent SEM. **(B)** Boxplots of pigeons' self-oriented behaviors in the experimental conditions (mean values are provided in Table 1). Condition had a significant effect on observed behaviors. Pigeons exhibited significantly more behaviors in the *Stranger* condition compared to the *Mirror* condition. The whiskers extend to the factor 1.5 of the interquartile range (IQR), with outliers omitted. *p < 0.05.

Activity rates were significantly different across conditions [Figure 2A, χ^2^(2) = 10.93, *p* = 0.004, η_*p*_^2^ = 0.07]. Subsequent multiple comparisons between the *Stranger* and the *Mirror* conditions revealed that the former produced higher activity rates than the latter (*z* = 3.31, pcorr = 0.003), but no difference was found between conditions *Mirror* and *Wall* (*z* = 1.90, pcorr = 0.14) and between conditions *Stranger* and *Wall* (*z* = 1.75, pcorr = 0.18). The comparison of the sessions within the conditions demonstrated that the activity patterns remained similar across the sessions in all conditions [*Stranger*: χ^2^(5) = 7.07, *p* = 0.22; *Mirror*: χ^2^(5) = 0.74, *p* = 0.98; *Wall*: χ^2^(5) = 0.74, *p* = 0.98].

Pigeons exhibited a number of other behaviors, as defined above (Table 1, shaking, preening, head scratching, wing flapping/opening, pecking Plexiglas/mirror/wall, and attack). A two-way repeated measures ANOVA revealed that condition [*F*_(2, 14)_ = 4.433, *p* = 0.032, η_*p*_^2^ = 0.388] and day [*F*_(5, 35)_ = 4.281, *p* = 0.004, η_*p*_^2^ = 0.380] had a significant effect on body-oriented behaviors (shaking, preening, head scratching, and wing flapping/opening), while their interaction was not significant [*F*_(10, 70)_ = 0.845, *p* = 0.588]. These behaviors occurred significantly more often in the *Stranger* as compared to the *Mirror* and the *Wall* conditions (Figure 2B, *Stranger* vs. *Mirror*: pcorr = 0.021; *Stranger* vs. *Wall:* pcorr = 0.022). The effect of the session was not significant within the conditions [*Stranger*: *F*_(5, 35)_ = 2.06, *p* = 0.09; *Mirror*: *F*_(5, 35)_ = 2.45, *p* = 0.053; *Wall*: *F*_(5, 35)_ = 1.22, *p* = 0.32]. Relative to the *Wall* condition, pigeons showed elevated surrounding-oriented behaviors (pecking Plexiglas/mirror/wall and attack) in *Stranger* and *Mirror* conditions, however these behaviors did not differ significantly among the conditions.

**Table 1:** Total and mean number of observed behaviors.

**Behaviors**	**Wall**			**Mirror**			**Stranger**		
	***sum***	***M***	***SEM***	***sum***	***M***	***SEM***	***sum***	***M***	***SEM***
Shaking	187	3.90	0.28	183	3.81	0.38	228	4.75	0.44
Preening	246	5.12	0.69	249	5.19	0.76	359	7.48	0.76
Head scratching	10	0.21	0.07	4	0.08	0.04	12	0.25	0.20
Wing flapping/opening	14	0.10	0.06	17	0.39	0.13	6	0.06	0.06
Pecking wall/mirror/plexiglas	0	0	0	9	0.19	0.08	21	0.44	0.16
Attack	0	0	0	12	0.25	0.17	10	0.21	0.11

## Discussion

Pigeons do not pass the classic MSR test in which animals have to spontaneously touch a mark on their body that is only visible in a mirror (Epstein et al., 1981; Uchino and Watanabe, 2014). Despite this fact, our study shows that pigeons do not equal their mirror reflection with a conspecific. We show that, in the *Mirror* condition, pigeons took more time to approach the mirror, exhibited a lower activity rate, less feeder/mirror-oriented behaviors and reduced amount of self-oriented activities than in the *Stranger* condition. These findings indicate that foraging pigeons spontaneously distinguish an unknown individual from one that behaves in perfect synchrony with themselves.

In nature, pigeons forage in groups (Lefebvre, 1985; Sol and Senar, 1995) and are very sensitive to dominance relationships during feeding (Diebschlag, 1940). Nagy et al. (2013) studied complex dominance hierarchies in groups of pigeons during social foraging. They calculated the dominance metric as the dot product of the velocity and distance vectors of one pigeon relative to another, with a positive value indicating a potentially dominant relationship. These two criteria can be viewed as functionally equivalent to the activity rate and the body orientation measure in our experiment. In the *Stranger* condition, we saw that these two components had a significantly higher value than in the *Mirror* condition. In the light of the study by Nagy et al. (2013), these results suggest that the presence of a stranger pigeon in the next compartment evoked a socially competitive foraging situation. Owing to the fact that the beak disappeared from the videos during most vertical foraging movement of the pigeons, it was not possible to track their beak, which might have provided direct information about head orientation. However, Theunissen and Troje (2017) demonstrated that pigeons often take a stable body position and compensate for the movement of environmental cues with head rotations. In the absence of moving cues, they usually keep their head-body position aligned. Therefore, tracking their beak should have provided no more information. In addition, the relationship between head orientation and vision field is complex in birds. They have very limited eye movements (Pratt, 1982) and move their head for better depth perception, using several specialized parts of their eyes (Stamp Dawkins, 2002). This question is out of the scope of the present study.

Birds spend around 9.2% of their daily time doing maintenance-related behaviors such as preening, scratching, and shaking (Cotgreave and Clayton, 1994). Lab and field studies show that foraging in front of a competitor represents a stressful situation, since the food consumed by the competitor is no longer available to oneself (Delius, 1967; Raouf et al., 2006). Indeed, when birds are exposed to a competitor or a potential predator, they show higher activity levels and especially elevated levels of preening and other kinds of displacement behaviors (Delius, 1967, 1988; Lima, 1995; Roberts, 1996; Fernández-Juricic et al., 2004). The same is true when they receive an injection of adrenocorticotropic hormone (ACTH) to induce stress (Delius et al., 1976; Kralj-Fiser et al., 2010) or an injection of dopamine (Acerbo, 2001). Indeed, the presence of a competitor in the next compartment increases the uncertainty to obtain food, a factor known to boost corticosterone and dopamine release in birds and mammals (e.g., Fiorillo et al., 2003; Hart et al., 2015; Marasco et al., 2015). It is premature to draw firm conclusions about the neurochemical systems involved in our behavioral study. What can be said, however, is that the higher rates of self-oriented behaviors in the *Stranger* condition (shaking, preening, scratching with the feet, wing flapping/opening) are also typically observed in wild pigeons and other avian species under conditions of behavioral conflicts, stress, food restriction, and social thwarting—i.e., under conditions often associated with the intrusion of a competitor (Delius, 1988; Henson et al., 2012).

Our findings show that pigeons displayed self-oriented (and other) behaviors significantly more in the *Stranger* than in the *Mirror* condition. Thus, pigeons are able to distinguish a real individual (potential competitor) and their mirror image (less credible competitor), for which there was more hesitation, in a social foraging situation.

It is unlikely that the significant effects in our results can be explained by the auditory cues induced by the other pigeon behind the Plexiglas barrier. First, activity rate was measured while the pigeons were not feeding, so the pecking sounds from the bird nearby could play no role. The differences in body orientation were also mainly observed after feeding, suggesting that pecking sounds played no role as well. Our conclusion that the effects obtained in this study result from visual inspection only are supported by findings in Clark's nutcrackers that show behavioral changes when confronted with a mirror image, independent of any potential noise or odor from a conspecific (Clary and Kelly, 2016).

Although pigeons do not show spontaneous MSR, we already mentioned that they can be conditioned for food reward to peck a spot on their body that is only visible in a mirror (Epstein et al., 1981; Uchino and Watanabe, 2014). Most important for the present study is also their ability to distinguish between live self-videos of themselves and pre-recorded self-videos that were taken in a previous session (Toda and Watanabe, 2008). Thus, pigeons can detect the kinesthetic correlation between mirror image and self-movements but seem to fail to infer its implication. Our study now pitched the mirror and the stranger situations as similar as possible to each other to be able to argue against the hypothesis of a sharp ‘cognitive Rubicon’. Instead, we argue that spontaneous MSR depends on at least two aptitudes: The first is the ability to detect the correlation between one's own movements and those in the mirror. The second is the cognitive capability to subsequently conclude that the mirror image is a reflection of oneself. Pigeons and some further species (de Waal et al., 2005; Desjardins and Fernald, 2010; Ueno et al., 2020) seem to manage the first ability but fail the second. These species are often disturbed by their mirror image and treat it like an uncanny conspecific but seem not to realize why this happens. Similarly, some brain-damaged human patients lose the ability to understand that a mirror reflects themselves and conclude that their mirror image is a strange person who happens to look similar to them (Feinberg, 2001).

Our results are in favor of a gradualist interpretation of MSR, where animal species can be placed along a continuum from a total absence of self-recognition (no synchronicity detection and no implication drawn) to full MSR (synchronicity detection and correct interpretation). A species whose members (or part of them) can detect synchronicity without drawing its major implication stands in an intermediate position. By failing to succeed in spontaneous MSR tasks while being able to detect movement synchronicity, pigeons, and probably many other species, are in this intermediate category of animals for which the mirror reflection is not another individual or themselves but something they cannot fully identify. It is likely that the gradual emergence of MSR is even more fine-grained since studies in macaques show that training can give rise to MSR (Chang et al., 2015, 2017). This gradualist explanation may make more sense from an evolutionary point of view than a sudden and parallel emergence of full-blown MSR in few branches of the animal kingdom (de Waal, 2019).

## Data Availability Statement

The datasets presented in this study can be found in online repositories. The names of the repository/repositories and accession number(s) can be found at: https://github.com/neslihanedes/avian-look-into-the-mirror-code and https://ruhr-uni-bochum.sciebo.de/s/WCL1Mgr3ITWmB5B.

## Ethics Statement

The animal study was reviewed and approved by All procedures followed the German guidelines for the care and use of animals in science, and were in accordance with the European Communities Council Directive 86/609/EEC concerning the care and use of animals for experimentation.

## Author Contributions

NW, OG, and PA designed the experiment. NK and NW tested the pigeons. NW, HM, and FO analyzed the results. NW, HM, PA, and OG wrote the manuscript. All authors gave final approval for publication.

## Conflict of Interest

The authors declare that the research was conducted in the absence of any commercial or financial relationships that could be construed as a potential conflict of interest.
